# Patients deriving long-term benefit from immune checkpoint inhibitors demonstrate conserved patterns of site-specific mutations

**DOI:** 10.1038/s41598-022-15714-5

**Published:** 2022-07-07

**Authors:** Daniel R. Principe

**Affiliations:** University of Illinois College of Medicine, 840 South Wood Street, 601 CSB, Chicago, IL 60612 USA

**Keywords:** Cancer, Oncology, Cancer, Tumour biomarkers, Tumour immunology

## Abstract

Immune checkpoint inhibitors (ICIs) have revolutionized cancer therapy and are now the preferred treatment for several tumor types. Though ICIs have shown remarkable efficacy in several cancer histologies, in many cases providing long-term disease control, not all patients will derive clinical benefit from such approaches. Given the lack of a reliable predictive biomarker for therapeutic responses to ICIs, we conducted a retrospective analysis of publicly available genomic data from a large pan-cancer cohort of patients receiving ICI-based immunotherapy. Consistent with previous results, patients in the combined cohort deriving a long-term survival benefit from ICIs were more likely to have a higher tumor mutational burden (TMB). However, this was not uniform across tumor-types, failing to predict for long-term survivorship in most non-melanoma cancers. Interestingly, long-term survivors in most cancers had conserved patterns of mutations affecting several genes. In melanoma, this included mutations affecting *TET1* or *PTPRD*. In patients with colorectal cancer, mutations affecting *TET1*, *RNF43*, *NCOA3*, *LATS1*, *NOTCH3*, or *CREBBP* were also associated with improved prognosis, as were mutations affecting *PTPRD, EPHA7*, *NTRK3*, or *ZFHX3* in non-small cell lung cancer, *RNF43, LATS1,* or *CREBBP* mutations in bladder cancer, and *VHL* mutations in renal cell carcinoma patients. Thus, this study identified several genes that may have utility as predictive biomarkers for therapeutic responses in patients receiving ICIs. As many have no known relationship to immunotherapy or ICIs, these genes warrant continued exploration, particularly for cancers in which established biomarkers such as PD-L1 expression or TMB have little predictive value.

## Introduction

Immune checkpoint inhibitors (ICIs) are a cornerstone of cancer therapy and have largely replaced broad-spectrum chemotherapy in several tumor types^1^. These strategies consist of antibodies directed against negative immune checkpoints including programmed cell death protein 1 (PD-1), PD-1 ligand 1 (PD-L1), and cytotoxic T-lymphocyte-associated protein 4 (CTLA-4). By disrupting the interaction between these molecules and their target proteins, these medications aim to de-restrain the cytotoxic immune program, thereby promoting immune-mediated destruction of tumor cells^2^. This approach has shown substantial anti-tumor activity in most cancer types, including melanoma, lung, and renal cancers^3–9^. Despite these successes, there remain many patients in ICI-sensitive tumors that fail to derive long-term clinical benefit from ICI-based immunotherapy^10^. As clinical responses can be highly varied even within the same tumor type, there is an increasing interest in identifying new, clinically useful biomarkers to predict for drug responses in patients receiving ICI-based immunotherapy^10^.

Several potential predictive biomarkers have been suggested, with PD-L1 expression earning FDA approval across several tumor types^11^. Despite this, PD-L1 status has shown only modest predictive value for ICI responsiveness, and many patients with PD-L1 non-expressing tumors will still show durable clinical benefit in response to ICI-based immunotherapy^11–13^. The FDA has also granted tissue agnostic approval for the PD-1 inhibitor Pembrolizumab for any solid tumor with high microsatellite instability (MSI-H) due to the presumed increase in DNA mismatches and larger tumor antigen pool, though recent estimates suggest that this applies to fewer than 4% of cancer patients^14–19^. Consistent with these observations, total mutational burden (TMB) has also been suggested as a potential means of predicting therapeutic responses across several tumor types^20,21^. Though TMB has shown promise in this application, TMB is still emerging as a predictive biomarker, and may be insufficient to predict for responses to ICIs alone^22,23^.

Given the lack of a reliable predictive biomarker, in this study we conducted a retrospective analysis of publicly available genomic data from a large pan-cancer cohort of patients receiving ICI-based immunotherapy. Consistent with previous observations in this cohort, patients with a high TMB demonstrated improved survival across the combined group^20^. However, we also demonstrate that mutations to select genes with no known relationship to cancer immunotherapy are also strongly associated with a long-term survival benefit, in some cohorts showing a more significant relationship than that between survival and TMB. Hence, several candidate genes identified in this study may have utility as an unrecognized predictive biomarker for drug responses and warrant continued exploration, particularly for cancers in which PD-L1 expression and TMB status demonstrate little predictive value.

## Methods

### Genomic database analysis

For the discovery cohort, the Sametein et al. patient dataset^20^ was downloaded and visualized using cBioPortal for Cancer Genomics as described in the original references^24,25^. Detailed information regarding the dataset, DNA sequencing analyses and protocols can be found in the original reference^20^. TMB was determined using the MSK-IMPACT assay also as described in the original reference^20^. Only patients receiving ICI-based immunotherapy were included, and all subsequent genetic analyses were restricted to fully sequenced tumors and gene sequences compared to a reference population as described in our previous studies^26–29^. For the melanoma validation cohort, the Van Allen et al., Snyder et al., and the Hugo et al. patient datasets were aggregated and analyzed as described. The non-small cell lung cancer (NSCLC) validation cohort consisted of the Riavi et al. and Hellmann et al. patient datasets, and the renal cell carcinoma (RCC) validation cohort the Miao et al. patient dataset. Additional information regarding these patients as well as^26–30^ DNA sequencing analyses and protocols can be found in the original references^31–36^. For the non-treated ICI cohort, the Zehir et al. patient dataset was downloaded and evaluated as described above. Additional information regarding this patient cohort, DNA sequencing analyses, and protocols can be found in the original reference^37^.

### Inclusion/exclusion criteria

All genomic analyses were restricted to fully sequenced tumors, and individualized cancer type analysis restricted to cohorts with an N ≥ 30. Analysis was restricted to patients receiving ICI-based immunotherapy in the form of an anti-PD-1, anti-PD-L1, and/or anti-CTLA-4 antibody unless otherwise stated.

### Statistical analysis

Patients were separated into groups based on final vital status, and differentially expressed genes were evaluated by an unpaired, non-parametric Mann–Whitney U-test and considered significant at an FDR-adjusted *P* value of < 0.05. Categorical data were evaluated using the Pearson’s chi-squared test. For time-to-endpoint/survival analyses, data were analyzed using the Kaplan Meier method/log rank test with hazard ratios and considered significant at a *P* value of < 0.05 as described previously^26–30^.

### Patient and public involvement statement

Neither our patients nor the public were involved in this study.

### Guide statement

All methods were carried out in accordance with relevant guidelines and regulations.

## Results

### Clinical characteristics of patients receiving immune checkpoint inhibitors

To identify additional prognostic biomarkers in patients receiving ICI-based immunotherapy, we evaluated the publicly available genomic data from the MSKCC pan-cancer cohort of 1661 patients, restricting analysis to patients receiving PD-1, PD-L1, and/or CTLA-4 inhibitors^20^. Of these 1661 patients, 11 cancer types were represented, including NSCLC (N = 350), melanoma (N = 320), bladder cancer (215), RCC (N = 151), head and neck cancer (N = 139), esophagogastric cancer (N = 126), glioma (N = 117), colorectal cancer (N = 110), cancer of unknown primary (N = 88), breast cancer (N = 44), and non-melanoma skin cancer (N = 1).

**Figure 1:**
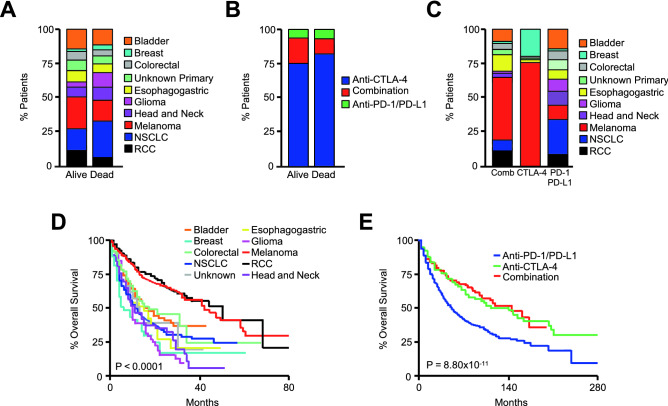
Clinical characteristics of patients receiving immune checkpoint inhibitors. (**A**) The Samstein et al. MSKCC pan-cancer cohort of 1661 patients receiving PD-1, PD-L1, and/or CTLA-4 inhibiting antibodies were arranged by vital status, and the tumor types represented in each group displayed by percent. (**B**) The percent of patients in each group receiving anti-PD-1/PD-L1 antibodies (N = 1307), anti-CTLA-4 antibodies (N = 99), or a combination of both (N = 255). (**C**) The percent of each tumor type arranged by treatment group. (**D**, **E**) Kaplan Meier plot showing overall survival for patients arranged by either cancer type or treatment group.

We subsequently arranged patients by overall vital status (alive or dead) and compared the clinical characteristics in each group using the Pearson's chi-squared test. In alive and dead groups, there was no statistically significant difference in natal sex or age group (Table S1). Additionally, in both alive and dead groups, all tumor types were similarly represented (Fig. 1A), and there was no significant difference between groups with respect to the immunotherapy regimen used (Fig. 1B). The immunotherapy approach used was closely related to tumor type, with the majority of patients receiving anti-CTLA-4 antibodies being treated for melanoma, with similar results observed in the combination (anti-PD-L1/PD-1 and anti-CTLA-4) group. Patients receiving anti-PD-L1/PD-1 were more diverse with all tumor types represented, though this group contained the majority of lung cancer patients (Fig. 1C). When arranged by the Kaplan Meier method, patients with melanoma and RCC had longer median overall survival when compared to all other cancers (Fig. 1D), and patients receiving anti-PD-L1 or anti-PD-1 had shorter overall survival when compared to those receiving anti-CTLA-4 or a combination regimen (Fig. 1E), likely attributed to the different cancer types represented within these groups.

### Mutations to genes associated with improved overall survival in the pan-cancer cohort

**Figure 2:**
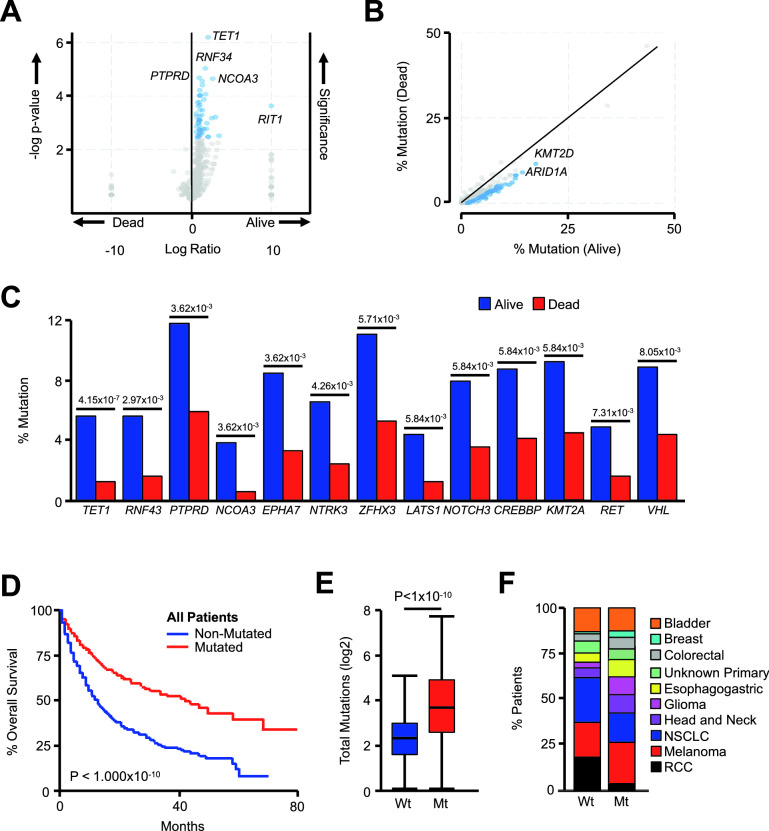
Mutations to genes associated with improved overall survival in the pan-cancer cohort. (**A**, **B**) Patients from the MSKCC pan-cancer cohort (N = 1661) were arranged by final vital status, and differentially mutated genes were arranged by volcano and scatter plot with those significantly different between the two groups shown in blue. (**C**) The 13 most differentially mutated genes are presented by percent mutated in each group, and shown with the corresponding false discovery rate (FDR)-adjusted P value. (**D**) Kaplan Meier plot showing overall survival for patients with or without a mutation in one or more of these 13 genes. (N = 1661 consisting of 610 patients with one or more mutation and 1051 non-mutated patients). (**E**, **F**) The total number of mutations and tumor types represented in patients with or without a mutation in one or more of these genes.

Using the described genomic data, we next evaluated the mutational profiles of patients in this study as arranged by vital status (alive or dead) using an unpaired, non-parametric Mann–Whitney U-test. This approach revealed several differentially mutated genes that were overrepresented in the alive group (Fig. 2A, B), with the 13 most significant (by FDR-adjusted P value) being *TET1*, *RNF43*, *PTPRD*, *NCOA3*, *EPHA7*, *NTRK3*, *ZFHX3*, *LATS1*, *NOTCH3*, *CREBBP*, *KMT2A*, *RET*, and *VHL* (Fig. 2C). The presence of a mutation to one of more of these genes was slightly more common in males compared to females, and these patients also had a marginal but statistically significant increase in median age (Table S2). The most frequent type of mutation in all genes was a missense mutation, many with presumed loss of function, though several other mutations were observed (Table S3). Patients with a mutation to one or more of these genes had a highly significant improvement in overall survival when compared to the non-mutated group (Fig. 2D), as well as an increase in the number of total mutations (Fig. 2E). Despite these differences, all 10 cancer types were represented in each group, though the non-mutated group had an increased frequency of RCC and NSCLC (Fig. 2F). Additionally, in the MSKCC pan-cancer cohort of 10,336 patients, the overwhelming majority of whom have not received ICI-based immunotherapy, there was no significant association between mutation to any of these genes and time-to-endpoint survival outcomes, with the exception of *VHL* which was associated with improved overall survival (Table S4). This was presumably because *VHL* mutations were observed almost exclusively in RCC tumors, which carry a better prognosis than other cancers included in this cohort. However, when restricting analysis to the 187 RCC patients in this cohort with clinical outcomes, there was no significant association between *VHL* mutation and clinical outcome (*P* = 0.258, 73 *VHL*-mutated and 114 *VHL*-non-mutated patients).

### *TET1* mutations are associated with improved outcomes in colorectal cancer and melanoma patients receiving immune checkpoint inhibitors

**Figure 3:**
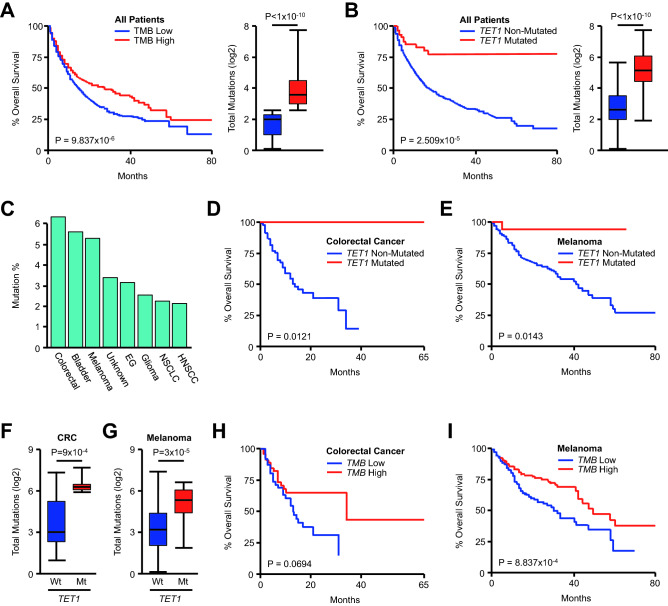
*TET1* mutations are associated with improved outcomes in colorectal cancer and melanoma patients receiving immune checkpoint inhibitors. (**A**) Kaplan Meier plot showing overall survival for the pan-cancer cohort (N = 1661) for patients with either a high (above median) or low (below median) tumor mutational burden (TMB). (**B**) Overall survival and the total number of mutations for all patients arranged by *TET1* mutation status (58 *TET1*-mutated and 1603 *TET1*-non-mutated patients) (**C**) The percent of *TET1*-mutated patients arranged by tumor type. (**D**–**G**) Overall survival and the total number of mutations for patients from the colorectal cancer (N = 110, 7 *TET1*-mutated and 103 *TET1*-non-mutated patients) or melanoma (N = 320, 18 *TET1*-mutated and 302 *TET1*-non-mutated patients) cohorts arranged by *TET1* mutation status. (**H**, **I**) Overall survival for the colorectal cancer and melanoma cohorts arranged by TMB.

To better explore the prognostic relevance of mutations to these genes, we first evaluated the survival for all patients arranged by TMB, to date the best prognostic factor established in this cohort^20^. Consistent with prior observations^20^, patients with a high (above the median) TMB had a significant survival benefit when compared to those with a low (below the median) TMB (Fig. 3A). *TET1* mutation was also strongly associated with improved survival across all patients, and *TET1*-mutated patients displayed a relative increase in TMB compared to *TET1*-non-mutated patients (Fig. 3B). As *TET1* mutations were most common in colorectal cancer, bladder cancer, and melanoma patients (Fig. 3C), we subsequently evaluated the relationship between *TET1* mutation and overall survival in these cancers specifically. Interestingly, *TET1* was a highly significant predictor of improved survival in colorectal cancer and melanoma patients (Fig. 3D, E), though there was no relationship between *TET1* mutation and survival in bladder cancer (*P* = 0.209). While *TET1* mutation was associated with an increase in total mutational burden in colon cancer and melanoma patients (Fig. 3F,G), the relationship between total mutational burden and survival did not reach statistical significance in this cohort of colorectal cancer patients (Fig. 3H), though it remained a significant prognostic factor in the melanoma cohort (Fig. 3I).

### *RNF43* mutations are associated with improved survival in colorectal and bladder cancer cohorts

**Figure 4:**
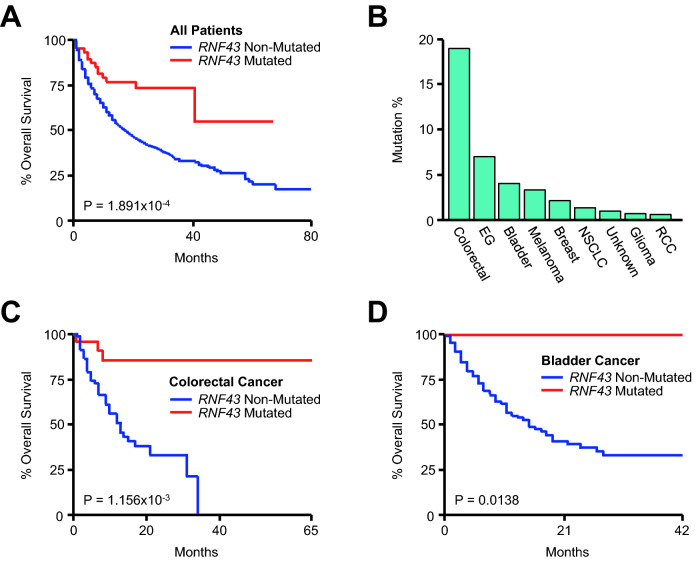
*RNF43* mutations are associated with improved survival in colorectal and bladder cancer patients receiving immune checkpoint inhibitors. (**A**) Kaplan Meier plot showing overall survival for the pan-cancer cohort (N = 1661) arranged by *RNF43* mutation status (61 *RNF43*-mutated and 1600 *RNF43*-non-mutated patients). (**B**) The percent of *RNF43*-mutated patients arranged by tumor type. (**C**, **D**) Overall survival for the colorectal (N = 110, 22 *RNF43*-mutated and 88 *RNF43*-non-mutated patients) and bladder cancer (N = 215, 9 *RNF43*-mutated and 206 *RNF43*-non-mutated patients) cohorts arranged by *RNF43* mutation status.

Given the relationship between *TET1* and clinical outcome, we next conducted a similar analysis using *RNF43*, the gene with the second strongest association with vital status in the pan-cancer cohort. Consistent with these observations, *RNF43* mutation was associated with improved survival across all patients using the Kaplan Meier method (Fig. 4A). *RNF43* mutation was most common in colorectal cancers, followed by esophagogastric and bladder cancers (Fig. 4B). *RNF43* mutations were strongly associated with improved overall survival in the colorectal and bladder cancer cohorts (Fig. 4C, D), surpassing the relationship between survival and total mutational burden in these patients (Fig. S1A). However, there was no significant relationship between *RNF43* mutation and overall survival in esophagogastric cancer patients (*P* = 0.504).

### *PTPRD* mutations are associated with improved survival in melanoma and lung cancer cohorts

**Figure 5:**
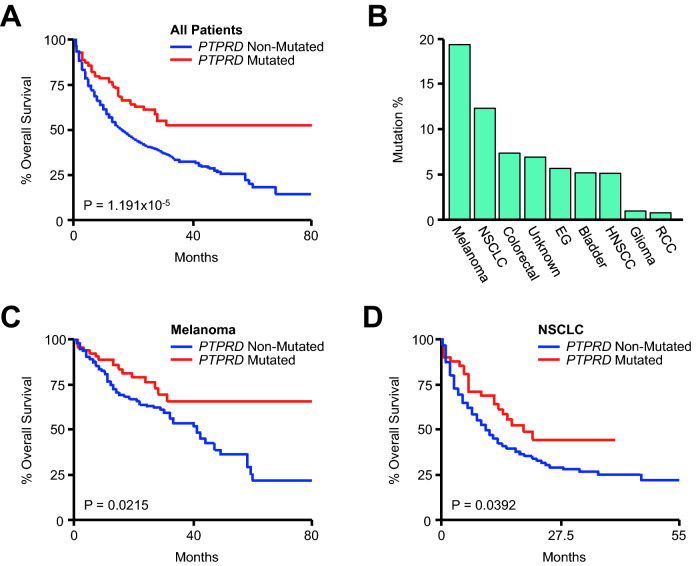
*PTPRD* mutations are associated with improved survival in melanoma and lung cancer patients receiving immune checkpoint inhibitors. (**A**) Kaplan Meier plot showing overall survival for the pan-cancer cohort (N = 1661) arranged by *PTPRD* mutation status (148 *PTPRD*-mutated and 1513 *PTPRD*-non-mutated patients). (**B**) The percent of *PTPRD*-mutated patients arranged by tumor type. (**C**, **D**) Overall survival for the melanoma (N = 320, 63 *PTPRD*-mutated and 257 *PTPRD*-non-mutated patients) and non-small cell lung cancer (NSCLC) (N = 350, 43 *PTPRD*-mutated and 307 *PTPRD*-non-mutated patients) cohorts arranged by *PTPRD* mutation status.

Continuing our analysis, *PTPRD* mutation status was also associated with improved overall survival in the pan-cancer cohort using the Kaplan Meier method (Fig. 5A). *PTPRD* mutations were most common in melanoma and NSCLC patients, followed distantly by colorectal cancer, cancers of unknown primary origin, esophagogastric cancer, bladder cancer, and HNSCC (Fig. 5B). *PTPRD* mutation was modestly associated with overall survival in melanoma and NSCLC cohorts (Fig. 5C, D), though this relationship was more significant than that between total mutational burden and overall survival (Fig. S1B). As *TET1* and *PTPRD* mutations were the only significant predictors of outcomes in the melanoma discovery cohort, we next explored their prognostic utility in a valuation cohort consisting of melanoma patients from three independent immunogenomic studies all receiving ICIs (N = 212). In this group, 23% of patients had a mutation in either gene, and as previously, *TET1*- or *PTPRD*-mutated patients had a highly significant improvement in overall survival compared to non-mutated patients, as well as a modest increase in TMB (Fig. S2).

### *NCOA3* and *NOTCH3* mutations are associated with improved survival in colorectal cancer patients

**Figure 6:**
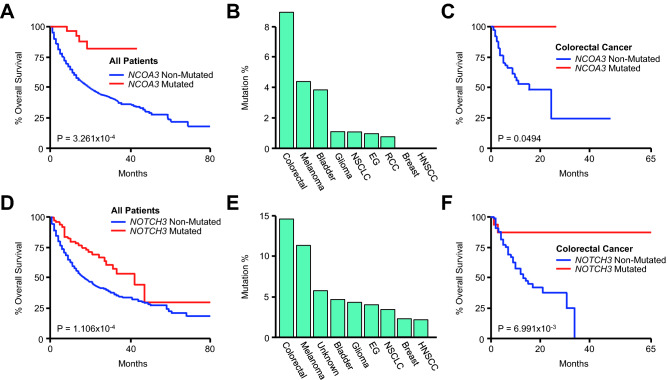
*NCOA3* and *NOTCH3* mutations are associated with improved survival in colorectal cancer patients receiving immune checkpoint inhibitors. (**A**) Kaplan Meier plot showing overall survival for the pan-cancer cohort (N = 1661) arranged by *NCOA3* mutation status (33 *NCOA3*-mutated and 1369 *NCOA3*-non-mutated patients. An additional 259 *NCOA3*-unprofiled patients were excluded from analysis). (**B**) The percent of *NCOA3*-mutated patients arranged by tumor type. (**C**) Overall survival for the colorectal cancer cohort arranged by *NCOA3* mutation status (N = 110, 8 *NCOA3*-mutated, 102 *NCOA3*-non-mutated patients). (**D**) Kaplan Meier plot showing overall survival for the combined pan-cancer cohort arranged by *NOTCH3* mutation status (96 *NOTCH3*-mutated and 1565 *NOTCH3*-non-mutated patients). (**E**) The percent of *NOTCH3*-mutated patients arranged by tumor type. (**F**) Overall survival for the colorectal cancer cohort arranged by *NOTCH3* mutation status (16 *NOTCH3*-mutated and 94 *NOTCH3*-non-mutated patients).

As previously, we next evaluated the prognostic relevance of *NCOA3* in the combined cohort using the Kaplan Meier method. Though *NCOA3* mutations were a favorable prognostic factor in the combined cohort (Fig. 6A), *NCOA3* mutations were predominant in colorectal cancer patients, followed by melanoma and bladder cancer patients (Fig. 6B). We subsequently evaluated the relationship between *NCOA3* mutation and survival in these cohorts and found that *NCOA3* mutation was associated with improved survival only in colorectal cancer, though this bordered on the threshold for statistical significance (Fig. 6C). We conducted a similar analysis using *NOTCH3*, which also was associated with improved survival in the pan-cancer cohort (Fig. 6D). *NOTCH3* mutation was also most frequent in colorectal cancers, followed by melanoma (Fig. 6E). However, *NOTCH3* mutation was only associated with improved survival in the colorectal cancer group, though unlike *NCOA3*, this relationship was highly significant (Fig. 6F).

### *EPHA7, NTRK3,* and *ZFHX3* mutations are associated with improved survival in lung cancer patients

**Figure 7:**
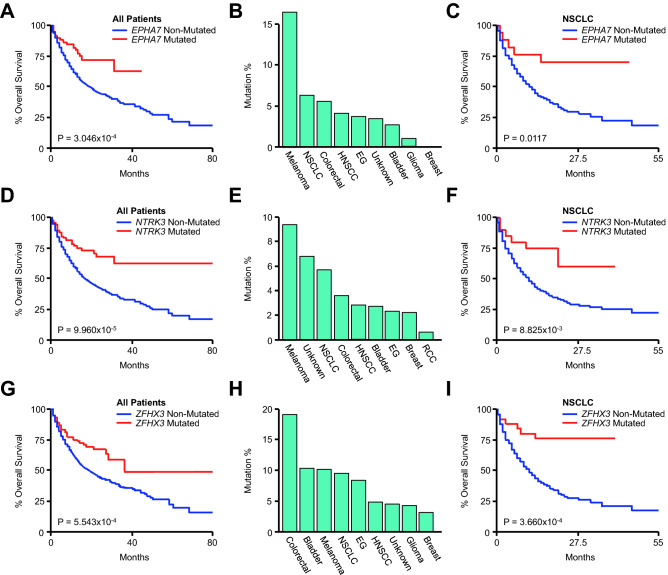
*EPHA7, NTRK3,* and *ZFHX3* mutations are associated with improved survival in lung cancer patients receiving immune checkpoint inhibitors. (**A**) Kaplan Meier plot showing overall survival for the pan-cancer cohort (N = 1661) arranged by *EPHA7* mutation status (86 *EPHA7*-mutated and 1316 *EPHA7*-non-mutated patients. An additional 259 *EPHA7*-unprofiled patients were excluded from analysis). (**B**) The percent of *EPHA7*-mutated patients arranged by tumor type. (**C**) Overall survival for the non-small cell lung cancer (NSCLC) cohort arranged by *EPHA7* mutation status (N = 350, 18 *EPHA7*-mutated and 265 *EPHA7*-non-mutated patients. An additional 67 *EPHA7*-unprofiled patients were excluded from analysis). (**D**) Kaplan Meier plot showing overall survival for the combined pan-cancer cohort arranged by *NTRK3* mutation status (76 *NTRK3*-mutated and 1585 *NTRK3*-non-mutated patients). (**E**) The percent of *NTRK3*-mutated patients arranged by tumor type. (**F**) Kaplan Meier plot showing overall survival for the NSCLC cohort arranged by *NTRK3* mutation status (20 *NTRK3*-mutated and 330 *NTRK3*-non-mutated patients). (**G**) Kaplan Meier plot showing overall survival for the pan-cancer cohort arranged by *ZFHX3* mutation status (118 *ZFHX3*-mutated and 1284 *ZFHX3*-non-mutated patients. An additional 259 *ZFHX3*-unprofiled patients were excluded from analysis). (**H**) The percent of *ZFHX3*-mutated patients arranged by tumor type. (I) Overall survival for the NSCLC cohort arranged by *ZFHX3* mutation status (27 *ZFHX3*-mutated and 256 *ZFHX3*-non-mutated patients. An additional 67 *ZFHX3*-unprofiled patients were excluded from analysis).

We next evaluated the relationship between overall survival and mutations to the next three genes identified, specifically *EPHA7, NTRK3,* and *ZFHX3.* Though *EPHA7* mutation was associated with improved survival in the combined cohort (Fig. 7A), *EPHA7* mutations were by far most frequent in melanoma patients, followed distantly by NSCLC and colorectal cancer patients (Fig. 7B). However, despite these observations, only *EPHA7* mutations had a statistically significant relationship with overall survival in NSCLC patients (Fig. 7C), failing to achieve statistical significance in any other cancer type. We observed similar results with respect to *NTRK3*, as *NTRK3* mutation was also associated with improved overall survival in the combined cohort (Fig. 7D), and was most frequent in melanoma patients, followed by cancers of unknown primary and NSCLC (Fig. 7E). However, *NTRK3* mutations only had a significant relationship with survival in NSCLC (Fig. 7F).

*ZFHX3* mutations were also associated with a significant survival benefit across all patients (Fig. 7G), and were most common in colorectal cancer patients, followed by bladder cancer, melanoma, and NSCLC patients (Fig. 7H). However, as previously, *ZFHX3* mutation was only a significant prognostic factor in NSCLC patients (Fig. 7I). As *EPHA7*, *NTRK3*, *ZFHX3*, and *PTPRD* mutations were all associated with improved outcomes in NSCLC patients in the discovery cohort, we next explored whether this relationship was also observed in a validation cohort consisting of 91 NSCLC patients also receiving ICIs. As observed in the discovery cohort, patients with a mutation in one or more of these genes had significantly longer progression-free survival compared to non-mutated patients (Fig. S3). Though overall survival data was not available, mutated patients also had a modest increase in TMB, consistent with observations from the discovery cohort (Fig. S3).

### *LATS1, CREBBP,* and *VHL* mutations are associated with improved survival in urologic malignancies

**Figure 8:**
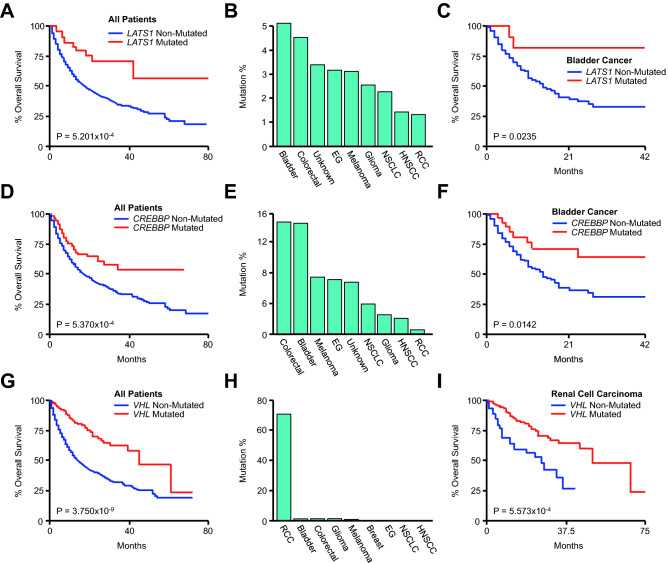
*LATS1, CREBBP,* and *VHL* mutations are associated with improved survival in urologic malignancies. (**A**) Kaplan Meier plot showing overall survival for the pan-cancer cohort (N = 1661) arranged by *LATS1* mutation status (48 *LATS1*-mutated and 1613 *LATS1*-non-mutated patients). (**B**) The percent of *LATS1*-mutated patients arranged by tumor type. (**C**) Kaplan Meier plot showing overall survival for the bladder cancer cohort arranged by *LATS1* mutation status (N = 215, 11 *LATS1*-mutated and 204 *LATS1*-non-mutated patients). (**D**) Kaplan Meier plot showing overall survival for the combined pan-cancer cohort method arranged by *CREBBP* mutation status (108 *CREBBP*-mutated and 1553 *CREBBP*-non-mutated patients). (**E**) The percent of *CREBBP*-mutated patients arranged by tumor type. (**F**) Kaplan Meier plot showing overall survival for the bladder cancer cohort arranged by *CREBBP* mutation status (31 *CREBBP*-mutated and 184 *CREBBP*-non-mutated patients). (**G**) Kaplan Meier plot showing overall survival for the pan-cancer cohort arranged by *VHL* mutation status (111 *VHL*-mutated and 1550 *VHL*-non-mutated patients). (**H**) The percent of *VHL*-mutated patients arranged by tumor type. (**I**) Kaplan Meier plot showing overall survival for the renal cell carcinoma cohort arranged by *VHL* mutation status (N = 151, 106 *VHL*-mutated and 45 *VHL*-non-mutated patients).

Finally, we explored the prognostic relevance of the remaining genes identified in our analysis. While *KMT2A* and *RET* mutations were associated with favorable overall survival in the combined cohort, there was no relationship between either *KMT2A* or *RET* mutation and survival in any individual cancer type (Fig. S4A–D). *LATS1* mutations were also strongly associated with improved survival in all patients (Fig. 8A) and were observed with similar frequency in several cancers including bladder cancer, colorectal cancer, cancers of unknown primary, esophagogastric cancer, melanoma, and others (Fig. 8B), though *LATS1* mutations were only associated with improved survival in bladder cancer and colorectal cancer patients (Figs. 8C and S5A). Similarly, *CREBBP* mutations were a positive prognostic factor in the combined cohort (Fig. 8D), and were most common in colorectal and bladder cancer patients (Fig. 8E). Accordingly, *CREBBP* mutations were strongly associated with improved survival in both bladder and colorectal cohorts (Figs. 8F and S5B). Though *VHL* mutations were also associated with improved overall survival in the combined cohort (Fig. 8G), *VHL* mutations were almost exclusive to RCC patients (Fig. 8H). Accordingly, in the RCC cohort, patients with a *VHL* mutation demonstrated a significant survival benefit compared to non-*VHL*-mutated patients (Fig. 8I), exceeding the predictive value of tumor mutational burden (Fig. S6).

We subsequently explored the relationship between *VHL* mutation and clinical outcomes in an independent validation cohort consisting of 35 clear cell RCC (ccRCC) patients also receiving ICIs. Though survival data was not available, *VHL*-mutated patients were more likely to derive clinical benefit from ICIs. In addition to being the only group with complete responses, 72% of *VHL*-mutated patients had at least an intermediate benefit from treatment compared to only 40% of non-mutated patients (Fig. S7).

## Discussion

ICIs have revolutionized cancer therapy in the past decade, and are now the preferred first-line treatment for several tumor types^1^. Though select patients will derive long-term clinical benefit from immune checkpoint inhibitors either as a monotherapy or in combination with other treatments, others will fail to show therapeutic responses or demonstrate only a transient response^38^. Hence, there is a clear need for novel, predictive biomarkers to identify the patients most likely to benefit from ICI-based immunotherapy, particularly given their often severe immune-mediated adverse effects^39^. As mentioned, patients with MSI-H tumors often show favorable response to ICIs, yet this is only relevant to a small fraction of cancer patients^14–19^. Additionally, while PD-L1 expression can be informative when predicting for responses to select ICIs such as Pembrolizumab, recent evidence suggests that PD-L1 status alone is not sufficient as a predictive biomarker and should be complemented by other clinical findings^11^. TMB is emerging as an important consideration in cancer immunotherapy, predicting for responses in a wide range of cancer types^20,21^. However, this is rather controversial, with other recent studies suggesting that, like PD-L1 status, TMB may have limited utility alone^22,23^.

In this study, we evaluated genomic data from a large pan-cancer cohort initially used to support the predictive value of TMB in patients receiving ICI-based immunotherapy. Consistent with the authors’ initial findings^20^, patients with a high TMB demonstrated a long-term survival benefit compared to those with a lower TMB. However, when retrospectively comparing mutation data from patients arranged by vital status at the study endpoint, we identified several genes that were differentially mutated in patients who derived a long-term clinical benefit from ICIs. Additionally, when restricting our analysis to patients with the same tumor types, though TMB failed to predict for long-term survivorship in many cohorts including colorectal cancer, several of these newly identified genes were strongly associated with improved outcomes.

For example, TET1 is a DNA demethylase belonging to the ten-eleven translocation family^40^. In the present study, we identified *TET1* mutation as a significant, positive predictor of outcomes in the combined cohort. Though *TET1* mutations were observed across a wide range of different cancers, *TET1* mutations were most strongly associated with improved responses to ICI-based immunotherapy in colorectal and melanoma patients, and in the case of colorectal cancer, exceeded the predictive value of TMB. This appears consistent with prior observations suggesting that *TET1* mutation may have utility as a predictive biomarker for ICIs. Recently, another study exploring predictors of therapeutic responses to ICIs in the Cancer Genome Atlas (TCGA) dataset also identified *TET1* mutation as a positive prognostic biomarker across the combined group. In the combined TCGA cohort, the authors reported that patients with a *TET1* mutation had a higher TMB, as observed in our study, as well as an increased neoantigen pool and an inflamed pattern of tumor-infiltrating immune signatures^41^. As our study also supports *TET1* mutation as a potential predictive biomarker, particularly for colorectal and melanoma patients, this warrants continued clinical investigation.

In addition to *TET1*, our study also identified mutations affecting the E3 ubiquitin-protein ligase *RNF43* as a potential predictor of therapeutic responses, notably for colorectal and bladder cancer patients. Classically, RNF43 functions as a negative regulator of oncogenic WNT/β-Catenin signaling and is considered a tumor suppressor gene^42^. However, several studies now also support an important immunomodulatory role for *RNF43*. For example, in a transgenic mouse model of early pancreatic ductal adenocarcinoma (PDAC), the loss of *RNF43* accelerated the formation of high-grade cystic lesions and led to extensive immune remodeling in the form of decreased macrophage infiltration, increased T- and B-cell infiltration, and enhanced expression of immune checkpoints. Further, these mice also appeared to derive benefit from CTLA-4 inhibition, with 2/6 showing a radiographic response and 4/6 showing stable disease^43^. Similar to our observations, *RNF43* mutations were associated with improved survival in colorectal cancer patients receiving ICIs in the TCGA cohort^44^. This is consistent with prior observations, also in colon cancer, where RNF43 was identified as a tumor-associated antigen that can elicit tumor-reactive cytotoxic T-cell responses^45^. Accordingly, RNF43 peptide-based therapeutic vaccines are now under investigation in combination with chemo- or immunotherapy in a variety of solid tumors^46–48^, and *RNF43* mutation status may also warrant exploration as a predictive biomarker.

In addition to *TET1* and *RNF43*, our study also identified an association between *NCOA3, CREBBP,* and *NOTCH3* mutations and improved survival in colorectal cancer patients. This is consistent with studies reporting a positive predictive role for *NCOA3* mutations in a multi-cancer cohort^49^, as well as *CREBBP* and *NOTCH3* mutations in the TCGA colorectal cohort^44^. Our study has also identified several additional candidate biomarkers in other tumor types, including *PTPRD* for melanoma and NSCLC patients. *PTPRD* is a tumor suppressor gene with important roles in regulating JAK/STAT signaling^50,51^. Though little is known regarding *PTPRD* mutation and drug responses in melanoma, previous observations support *PTPRD* mutations (or those to the functionally related gene *PTPRT*) as a positive predictor of outcomes in NSCLC patients receiving ICIs in a pooled cohort from three publicly available datasets as well as the TCGA cohort of NSCLC patients^52^. Our study additionally identified a similar role for *EPHA7* and *ZFHX3* mutations in NSCLC, consistent with prior observations from a large pan-cancer cohort^49,53^.

Though there is limited data supporting mutations to the genes described above as potentially useful biomarkers, our study identified several other candidate genes that have yet to be suggested in this application. While *NTRK3* mutations have yet to be linked to response to ICIs, there is early evidence that *NTRK3*-mutated bladder cancers are more immunogenic, with increased TMB and enhanced immune infiltration compared to non-mutated tumors^54^. Similarly, while our study determined that *LATS1* mutations were also associated with a survival benefit in the combined, bladder, and colorectal cancer cohorts, very little is known regarding *LATS1* mutations and ICI responses. However, *Lats1/2* deletion led to regression of three murine syngeneic tumor models. Further, this required the adaptive immune system, and *Lats1/2* deficiency enhanced the efficacy of tumor vaccines *in vivo*^55^. Similarly, though RET is not a known biomarker in immune oncology, RET does appear to have central roles in directing the tumor microenvironment, promoting cancer-associated inflammation, and suppressing anti-tumor immune responses^56^. Hence, loss of function mutations to genes such as *LATS1 or RET* may lead to considerable immune reprogramming, explaining the improved outcomes in patients receiving ICIs in this study.

Finally, our study identified a highly significant association between *VHL* mutation and improved survival in RCC patients. *VHL* is a tumor suppressor gene often mutated in ccRCC patients^57^. At the present time, *VHL* mutations are not known to confer increased sensitivity to ICIs in RCC patients. However, *VHL* mutations confer increased sensitivity to natural killer cell-mediated killing of RCC cells *in vitro*^58^, with similar observations in RCC patients^59^. Hence, as ICIs are a cornerstone in the treatment of RCC^60^, this observation warrants investigation. This can be said for the several candidate genes identified in this study, many of which may have utility as unrecognized biomarkers for drug responses in patients receiving ICIs. However, it is important to recognize the inherent limitation of public database analyses. For example, though these data are compelling, these patient datasets do not fully account for additional factors including demographic data, cancer stage, line of treatment, comorbidity profile, performance status, and others. Hence, these should be included in future prospective analyses before any of these candidate biomarkers are used to guide clinical decision making. Similarly, though several mutations identified presumably confer loss of function, others are of unknown significance. Hence, this too warrants exploration in a laboratory setting. However, given the highly varied responses in patients receiving these medications, these and other biomarkers warrant continued exploration, particularly for cancers in which better-established methods such as PD-L1 expression and TMB fail to reliably predict for patients most likely to derive clinical benefit from ICI-based immunotherapy.

## Supplementary Information

Supplementary Information.

## Data Availability

The datasets analyzed in the current study are publicly available and can be found at www.cBioPortal.org.
