# Supplementary material for: Patients deriving long-term benefit from immune checkpoint inhibitors demonstrate conserved patterns of site-specific mutations
Source: Sci Rep. 2022 Jul 7;12:11490. doi: 10.1038/s41598-022-15714-5 (PMC9263148; doi:10.1038/s41598-022-15714-5)
Supplement: Supplementary file 1 — Supplementary Information. [file 41598_2022_15714_MOESM1_ESM.pdf]

Patients Deriving Long-Term Benefit from Immune Checkpoint Inhibitors Demonstrate Conserved Patterns of Site-Specific Mutations

Daniel R. Principe[1]

[1]University of Illinois College of Medicine, Chicago, IL

Short Title: Mutations associated with durable response to immune checkpoint inhibition

Pages	26
Tables	4
Figures	7
Words	862

Correspondence to:

Daniel R. Principe
University of Illinois College of Medicine
840 South Wood Street, 601 CSB
Chicago, IL 60612
Tel: (312) 413-7271
Email: principe@illinois.edu

Conflict of Interest Disclosure: The author has no conflicts to disclose.

SUPPLEMENTAL TABLES

Categorical Variable	N	Median OS (Months)	Log Rank Test P Value
**Natal Sex**			0.0722
Male	1034	15.00	
Female	324	19.00	
**Age Group**			0.979
Under 60	912	19.00	
Over 60	749	17.00	

Table S1. Overall survival for patients receiving immune checkpoint inhibitors arranged by either age or natal sex

	Mutated (N=610)	Non-Mutated (N=651)	Log Rank Test P Value
**Natal Sex**			0.0162
Male	404 (66.23%)	630 (59.94%)	
Female	206 (33.77%)	421 (40.06%)	
**Median Age**	64	62	1.389x10-5

Table S2. Demographic data for patients with or without an alteration in the 13 gene panel in the immune checkpoint inhibitor cohort

Gene	Total Mutations	Missense	Nonsense	FS Insertion	FS Deletion	IF Insertion	IF Deletion	Splice	Fusion
**TET1**	75	59	8	-	6	-	-	1	1
**RNF43**	79	35	9	2	29	2	1	2	1
**PTPRD**	201	176	14	3	1	-	-	6	1
**NCOA3**	33	23	2	-	4	3	-	-	1
**EPHA7**	109	98	4	-	1	-	-	6	-
**NTRK3**	93	84	2*		2			4	1
**ZFHX3**	144	114	8	3	11	1	4	-	3
**LATS1**	52	38	7	3	3	-	1	-	-
**NOTCH3**	119	98	5	2	7	-	-	4	3
**CREBBP**	131	86	14	6	16	-	2	5	2
**KMT2A**	155	116	18	4	11	-	2	2	2
**RET**	58	50	6	-	1	-	-	-	1
**VHL**	111	42	15	16	23	-	3	12	-

Table S3. Frequency and type of mutations associated with improved survival in the pan-cancer cohort of patients receiving immune checkpoint inhibitors

Abbreviations: Frameshift (FS); Inframe (IF); *Includes a nonstop truncating mutation

Gene	N	Median OS (Months)	Log Rank Test P Value
TET1-Mutated	200	30.24	0.726
TET1-Non-Mutated	7,357	26.20	
RNF43-Mutated	221	30.47	0.745
RNF43-Non-Mutated	7,335	26.20	
PTPRD-Mutated	413	26.04	0.204
PTPRD-Non-Mutated	7,138	26.20	
NCOA3-Mutated	103	N/A	0.182
NCOA3-Non-Mutated	5,724	26.56	
EPHA7-Mutated	207	N/A	0.270
EPHA7-Non-Mutated	5,622	26.56	
NTRK3-Mutated	222	21.79	0.268
NTRK3-Non-Mutated	7,337	26.20	
ZFHX3-Mutated	303	N/A	0.789
ZFHX3-Non-Mutated	5,528	26.56	
LATS1-Mutated	148	28.44	0.829
LATS1-Non-Mutated	7,411	26.13	
NOTCH3-Mutated	303	24.03	0.817
NOTCH3-Non-Mutated	7,263	26.23	
CREBBP-Mutated	358	25.74	0.793
CREBBP-Non-Mutated	7,194	26.20	
KMT2A-Mutated	287	28.73	0.985
KMT2A-Non-Mutated	7,272	26.13	
RET-Mutated	185	21.79	0.189
RET-Non-Mutated	7,375	26.20	
VHL-Mutated	102	N/A	**0.038**
VHL-Non-Mutated	7,466	26.13	

Table S4. Mutations to genes associated with improved survival in the pan-cancer cohort receiving immune checkpoint inhibitors do not predict for improved survival in patients not receiving immunotherapy

Overall survival of the MSKCC pan-cancer cohort (N=10,336), the majority of whom have not received immunotherapy, arranged by mutation status to the 13 prognostic genes identified in the immune checkpoint inhibitor cohort.

Figure S1

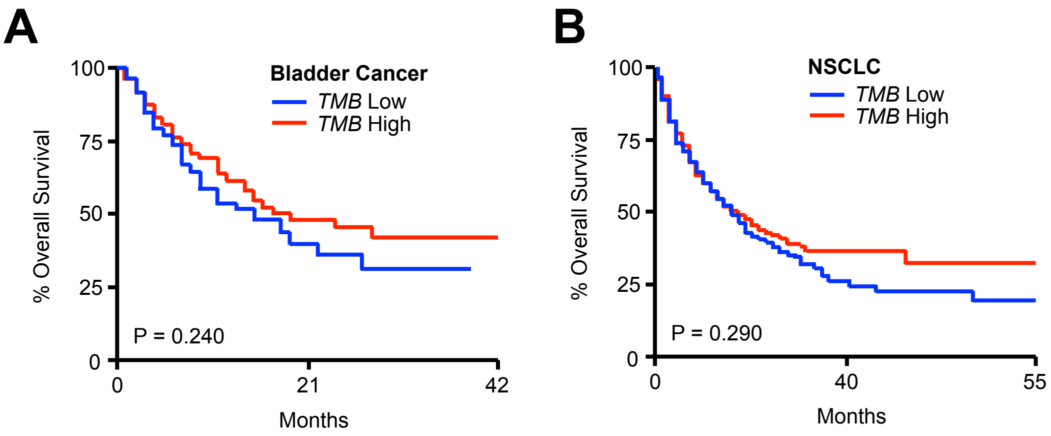

Figure S1. Tumor mutational burden does not predict for overall survival in the bladder and lung cancer cohorts

Kaplan Meier plot showing overall survival for patients with either a high (above median) or low (below median) tumor mutational burden (TMB) in the **(A)** bladder cancer cohort (N=215) or the **(B)** non-small cell lung cancer (NSCLC, N=350) cohorts.

Figure S2

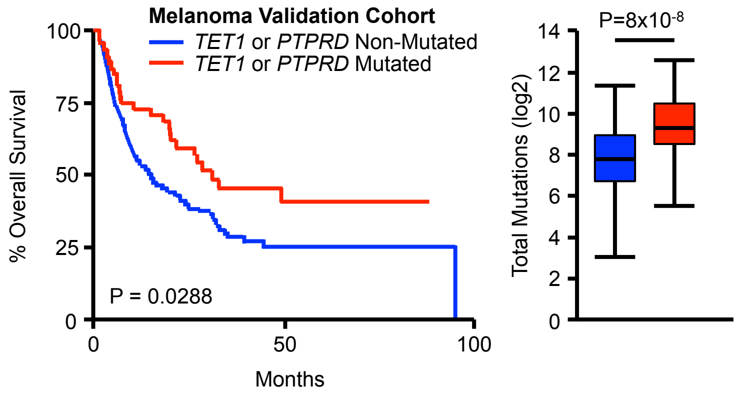

Figure S2. *TET1* and/or *PTPRD* mutations are associated with improved overall survival in an independent, validation cohort of melanoma patients
Overall survival for melanoma patients from three independent immunogenomic studies receiving ICIs (N=212) arranged by *TET1* and/or *PTPRD* mutation status (61 mutated and 151 non-mutated patients).

Figure S3

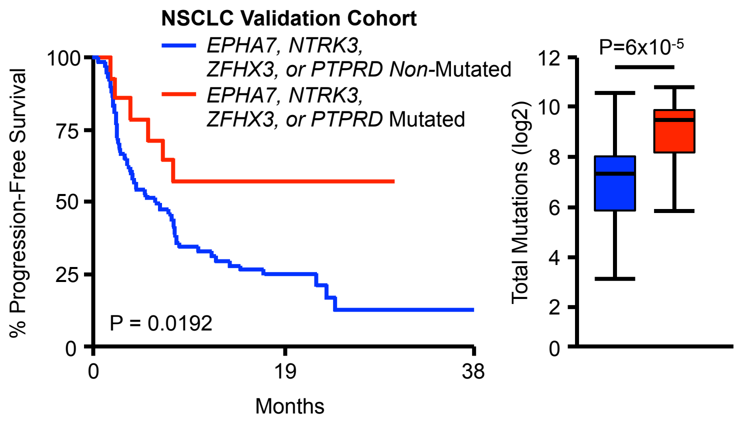

Figure S3. ***EPHA7, NTRK3, ZFHX3,*** **and/or** ***PTPRD*** **mutations are associated with improved overall survival in an independent, validation cohort of lung cancer patients**

Overall survival for non-small cell lung cancer (NSCLC) patients (N=91) receiving ICIs arranged by *EPHA7, NTRK3, ZFHX3,* and/or *PTPRD* mutation status (15 mutated and 76 non-mutated patients).

Figure S4

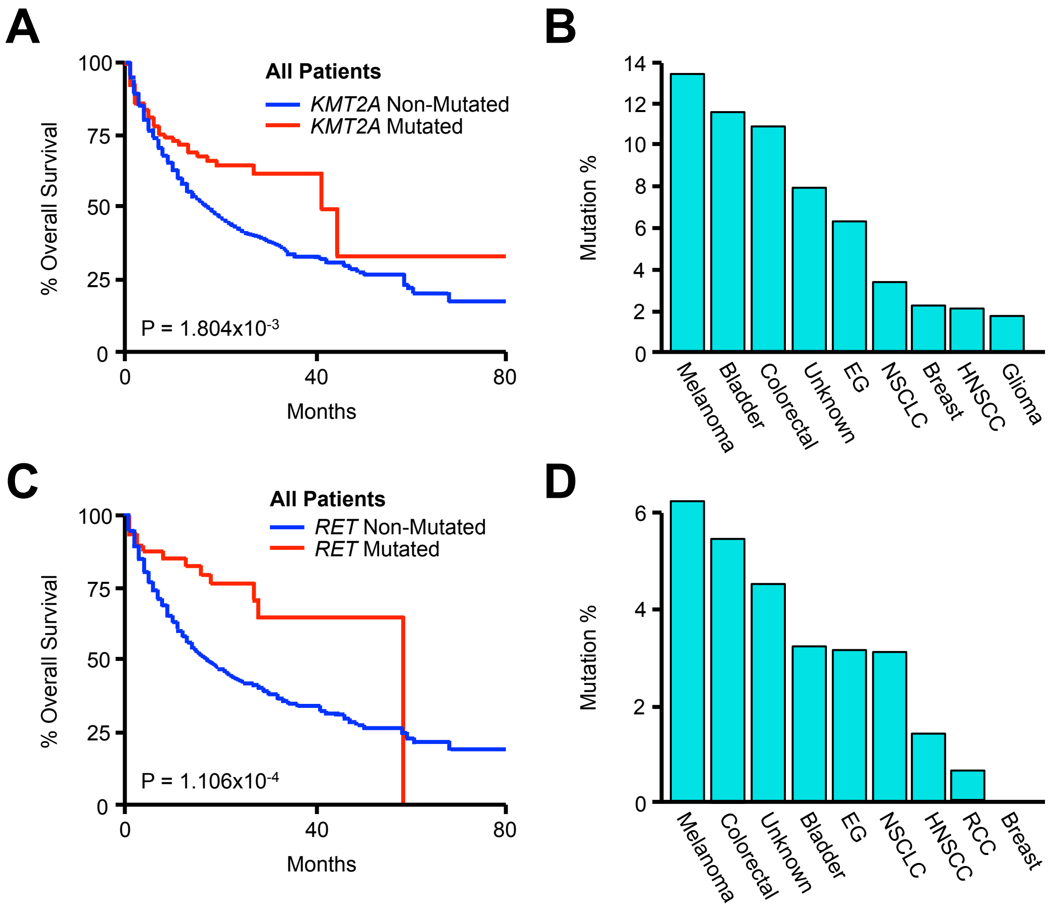

Figure S4. *KMT2A* and *RET* mutations are associated with improved overall survival in the combined pan-cancer cohort

(A) Overall survival of the pan-cancer cohort (N=1,661) displayed by the Kaplan Meier method arranged by *KMT2A* mutation status (115 *KMT2A*-mutated and 1,546 *KMT2A*-non-mutated patients). **(B)** The percent of *KMT2A*-mutated patients arranged by tumor type. **(C)** Overall survival of the pan-cancer cohort (N=1,661) displayed by the Kaplan Meier method arranged by *RET* mutation status (55 *RET*-mutated and 1,606 *RET*-non-mutated patients). **(D)** The percent of *RET*-mutated patients arranged by tumor type.

Figure S5

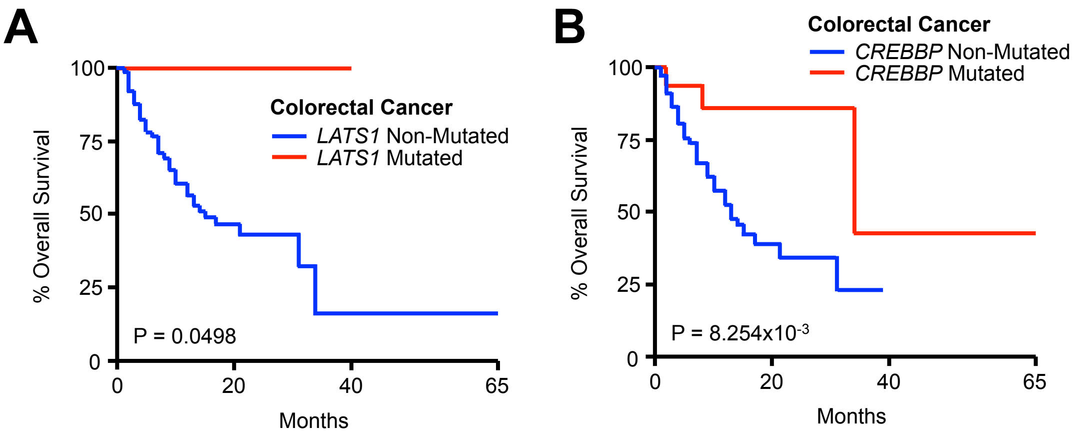

Figure S5. LATS1 and CREBBP mutations are associated with improved overall survival in the colorectal cancer cohort

(A,B) Overall survival for the colorectal cancer cohort (N=110) arranged by LATS1 (5 LATS1-mutated and 105 LATS1-non-mutated patients) or CREBBP (16 CREBBP-mutated and 94 CREBBP-non-mutated patients) mutation status.

Figure S6

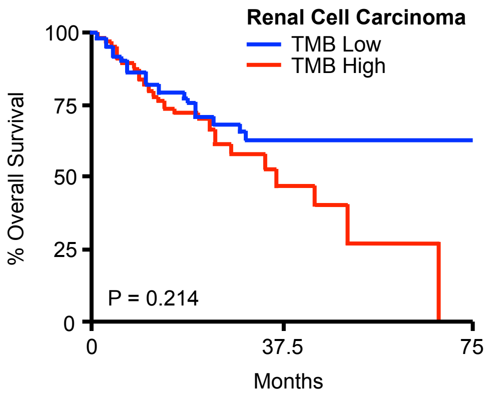

Figure S6. Tumor mutational burden does not predict for improved overall survival in the renal cell carcinoma cohort
Overall survival for patients from the renal cancer carcinoma (N=151) with either a high (above median) or low (below median) tumor mutational burden (TMB).

Figure S7

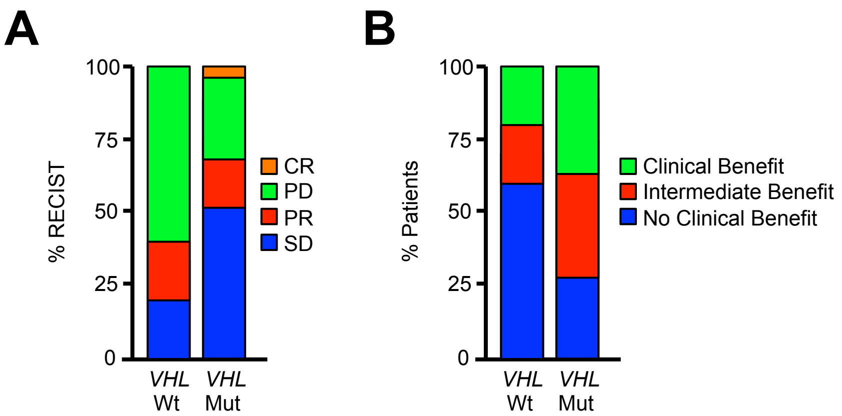

Figure S7. VHL-mutated renal cancer carcinoma patients are more likely to derive clinical benefit from immune checkpoint inhibition in an independent validation cohort
(A) The percent of patients experiencing a complete response (CR), partial response (PR), stable disease (SD), or progressive disease (PD) in the validation cohort of clear cell renal cell carcinoma patients being treated with immune checkpoint inhibitors (N=35) arranged by *VHL* mutation status (25 *VHL*-mutated and 10 *VHL*-non-mutated patients). **(B)** The percent of patients in this cohort experiencing either a clinical benefit, intermediate benefit, or no clinical benefit arranged by *VHL* mutation status.
